# Onco-nephrology in kidney transplant recipients: challenges and evolving strategies

**DOI:** 10.3389/fimmu.2026.1778752

**Published:** 2026-07-14

**Authors:** Carlo Alfieri, Mariadelina Simeoni, Margherita Di Naro, Deborah Mattinzoli, Masami Ikehata, Silvia Armelloni, Paolo Molinari, Marco Maggi, Alberto Mella, Anna Regalia, Simona Verdesca, Evaldo Favi, Laura Cosmai, Piergiorgio Messa, Giuseppe Castellano

**Affiliations:** 1 Unit of Nephrology, Dialysis and Kidney Transplantation, Fondazione IRCCS Ca’ Granda Ospedale Maggiore Policlinico di Milano, Milan, Italy; 2 Department of Clinical Sciences and Community Health, Dipartimento di Eccellenza, University of Milan, Milan, Italy; 3 Department of Translation Medical Sciences, University of Campania “Luigi Vanvitelli”, Naples, Italy; 4 Renal Research Laboratory, Fondazione IRCCS Ca’ Granda Ospedale Maggiore Policlinico, Milan, Italy; 5 Division of Nephrology Dialysis and Renal Transplantation - “Città della Salute e della Scienza” University Hospital, Turin, Italy; 6 General Surgery and Kidney Transplantation, Fondazione IRCCS Ca’ Granda Ospedale Maggiore Policlinico, Milan, Italy; 7 UOC Nephrology and Dialysis, Azienda Socio-Sanitaria Territoriale (ASST) Fatebenefratelli-Sacco, Fatebenefratelli Hospital, Milan, Italy

**Keywords:** cancer, immune checkpoint inhibitors, kidney transplant, management, neoplasia

## Abstract

Cancer is a significant cause of mortality in kidney transplant recipients (KTr). Specifically, the relationship between cancer and kidney transplantation (KTx) is evident from the initial evaluation of CKD patients prior to their placement on the KTx waiting list. After KTx, a significant impact on the potential development of cancer is linked to immunosuppressive therapies, necessary to maintain renal function post-KTx, and to complications, especially infectious ones, typical of these patients. Ultimately, once cancer is diagnosed in a KTx, nephrologists and transplant specialists are forced to modulate suppressive therapies and, together with oncologists, manage specific cancer treatment therapies. In this highly complex scenario, unfortunately, there are few certainties, and they are limited to a few studies, often involving a small number of patients. This review integrates current data on risk, prevention, and treatment across the entire pathway: pre-transplant assessment, post-transplant surveillance, and management after a cancer diagnosis. A special focus will be reserved to the new evidences regarding the treatment with immune-checkpoint inhibitors (ICIs).

## Introduction

Today, neoplastic disease represents a significant challenge in the management of solid organ transplant recipients in general, and especially kidney transplant recipients (KTxr). Cancer represents a significant risk of morbidity and mortality both short and long-term (1). Data reported in literature indicate that, unlike infectious and cardiovascular diseases, the mortality rate associated with cancer is particularly high today and has been increasing over the years in KTxr (2).

KTxr are unique patients in the population of neoplastic patients. In the setting of kidney transplantation (KTx) in fact, three moments that strongly influence the relationship between cancer and KTx can be recognized. The first stage involves the careful assessment of the presence of neoplasms when evaluating transplant donors, which is of fundamental importance. Furthermore, there is growing evidence today of patients being listed for KTx waiting list who have had a history of neoplastic diseases. In a second phase, once the transplant has been performed, the issue of post-transplant clinical and therapeutic approaches becomes evident, as well as the degree of immunosuppression and the development of infectious complications, especially viral ones, which can lead to the development of neoplasms in the long term. A final, absolutely important point concerns the management of patients who develop a neoplasm after transplantation. There are two key points to consider here: the modulation of immunosuppressive therapy, and, of course, the specific oncology therapy, which requires close collaboration between treating nephrologists and oncology colleagues.

This narrative review aims to provide a critical and clinically informed summary of the main oncological issues in renal transplantation, considering the entire patient journey: from the evaluation of candidates with prior or potential cancer, to post-transplant oncological surveillance, to the therapeutic management of recipients who develop cancer. Particular attention is paid to the most practical aspects—risk stratification, screening, monitoring, modulation of immunosuppression, and multidisciplinary decision-making—as well as emerging evidence on the use of immune checkpoint inhibitors in renal transplant recipients.

## Methods and characteristics of the review

This review was designed as a narrative review. We searched PubMed/MEDLINE for English-language articles using combinations of the terms ‘kidney transplantation’, ‘cancer’, ‘malignancy’, ‘immune checkpoint inhibitors’, ‘belatacept’, ‘mTOR inhibitors’, ‘screening’, ‘post-transplant lymphoproliferative disorder’, and ‘donor-transmitted cancer’. Priority was given to clinical practice guidelines, consensus statements, prospective studies, registry-based analyses, and clinically relevant translational studies. Additional references were identified through manual review of the bibliographies of selected articles (3).

## Evaluation of the patient eligible for kidney transplant with previous cancer

Evaluating the patient eligible for transplant, with previous cancer, is certainly the first crucial point to address. Recent literature data published by Benjamin et al. have reported a close correlation between chronic kidney disease and cancer risk. Specifically, these authors conducted a meta-analysis to compare the incidence of cancer in patients with eGFR <60 vs. ≥60 mL/min/1.73 m², also exploring subjects with eGFRs between 60 and 89 mL/min and ≥90 mL/min. Patients with eGFR <60 mL/min had a significantly higher incidence of cancer than those with normal or only mildly reduced kidney function, with age also contributing. This clearly demonstrates the strong relationship between kidney disease and cancer and how it is something that can’t be ignored in the clinical practice, as is the relationship with age, given that we are currently accepting increasingly older patients on the transplant lists. CKD may promote a pro-oncogenic milieu through chronic inflammation, oxidative stress, immune dysfunction, uremic toxin accumulation, microbiota changes, and impaired DNA repair, while aging likely amplifies this association (4, 5). Over the years, several guidelines have been developed with the aim of providing more detailed indications regarding the approach to be taken when evaluating patients for KTx. In particular, it is important to remember that the timing of placing patients with a history of cancer on the waiting list must take into account various factors, which include the stage at diagnosis, the time elapsed since the last treatment, the response to the treatment, the type of transplant to which the patients must undergo and clearly the level of immunosuppression to which they must be subjected. Unlike hematopoietic transplantation, kidney transplantation currently lacks a universally validated cancer-specific pretransplant risk score. Therefore, listing decisions should rely on individualized multidisciplinary assessment integrating stage, grade, disease-free interval, response to treatment, tumor biology, and expected immunosuppressive burden (6). There is the need today in the evaluation of patients previously affected by hematological diseases or solid neoplasia of a strict collaboration with hematologists and oncologists (7, 8). Specifically, beyond the current timescales that are based solely on the degree of neoplasia, it is possible to improve the estimation of actual neoplastic risk by also incorporating the biomolecular characteristics of each individual tumor (9). Very recently, a working group of transplant experts, together with oncology experts, attempted to determine the optimal timing of transplantation for patients with a history of cancer treated before KTx. They emphasized the urgent need to initiate and support prospective studies that validate specific cancer surveillance models for transplant recipients, to improve the clinical management of this high-risk population (10). However, the real question is whether a history of pretransplant cancer can be considered a risk factor for the development of cancer posttransplant and for the general mortality of the patients: this is certainly a crucial issue for candidate selection. In the recently published study by Hart et al., involving more than 300000 patients, the impact of a pretransplant cancer diagnosis on mortality in solid organ transplant recipients was investigated. The presence of a single pretransplant cancer was associated with an increase in both overall mortality (aHR 1.19; 95% CI 1.15–1.23) and cancer-specific mortality (aHR 1.93; 95% CI 1.76–2.12), with similar results for two or more pretransplant cancers. Analysis by tumor site showed marked increases for lung cancer (aHR 3.72) and multiple myeloma (aHR 4.42), while there were no significant differences for uterine, prostate, or thyroid cancers. Furthermore, a prior malignancy was associated with a higher risk of developing a *de novo* cancer after transplant (aHR 1.32; 95% CI 1.23–1.40). Among patients who died of cancer with data confirmed by oncology registries, over half (51.6%) died of a new post-transplant tumor, while approximately one third (34.3%) died of a recurrence of the pre-transplant tumor (11).

### The reception of a kidney from a donor with a neoplasm

In the pre-transplant period, it is important to keep in mind a rare, yet possible, event: receiving a kidney from a donor with a neoplasm. This is a topic addressed by two interesting studies recently published in the literature. The first collected a series of case reports and identified the most frequently transmitted tumors as lymphoma (20.5%), renal cell carcinoma (17.9%), and melanoma (17.1%), followed by non-small cell lung cancer (5.6%) and neuroendocrine tumors, including small cell lung cancer (4.7%) and choriocarcinoma (4.3%). Glioblastoma and gastrointestinal neoplasms were frankly rare. Prognosis varied significantly by histotype: melanoma and lung cancer had the worst survival (5-year overall survival of 43% and 19%, respectively), while renal cell carcinoma and lymphomas showed more favorable outcomes (5-year overall survival of 93% and 63%). Extra graft metastasis was the main adverse prognostic factor (12). Mahíllo et al, analyzed the Spanish experience (2013–2018) on the risk of donor-to-recipient tumor transmission in transplants. Of a total of 10076 deceased donors used, 349 (3.5%) had been diagnosed with a malignancy: 275 with a history or known ongoing disease before transplantation (for a total of 651 recipients) and 74 diagnosed only after transplantation. Among donors diagnosed after transplantation, among 126 at-risk recipients, only 10 donors actually transmitted a tumor to 16 recipients, with various etiologies: lung cancer, duodenal cancer, renal cell carcinoma, extrahepatic cholangiocarcinoma, prostate cancer, and undifferentiated neoplasia. After a median follow-up of 14 months, 9 of the 16 recipients died. Overall, among 802 at-risk recipients, transmitted tumors developed in 16 cases (2%), corresponding to approximately 6 cases per 10,000 transplants. All these data indicate that the current exclusion criteria may be excessively cautious, opening the possibility of a more personalized risk assessment, capable of expanding the donor pool without compromising the safety of the recipients (13).

## The influence on cancer risk of the post-transplant management

Post-transplant, several factors can impact the short- and long-term onset of cancer. Immunosuppressive therapy, viral infections, and other complications typical of the transplant state play a key role in increasing the risk of cancer.

Renal transplant recipients require lifelong immunosuppressive therapy to maintain optimal graft function. However, while essential for preventing rejection, this therapy inevitably impairs immune surveillance and, together with other direct effects, can substantially increase the risk of cancer development (14). Furthermore, the viral activation often observed in these patients, particularly EBV, HCV, HHV8, and HPV, can play a significant trigger in the oncogenic process (15). An impact on cancer risk can be determined as early as the induction therapy schedule. In the work by Crepin et al, the effect of immunosuppressive induction with anti-thymocyte globulin (ATG) was compared with Basiliximab on various parameters related to immune senescence (a key factor in oncogenic processes) in renal transplant recipients. Both ATG and Basiliximab are drugs that are currently used in the induction phase. Basiliximab is a chimeric monoclonal antibody that prevents rejection by selectively binding to the interleukin-2 receptor (CD25) on activated T lymphocytes. This binding blocks the action of IL-2, inhibiting T lymphocyte proliferation, thus reducing the immune response against the transplanted organ (16). ATG act inducing a rapid and marked depletion of circulating T lymphocytes that are the main responsible for rejection (17). Biomarkers such as thymic production estimated by T-cell receptor excision circles or by quantification of recent thymic emigrants, frequency of hematopoietic progenitors, T cell phenotype, telomere length, and telomerase activity were analyzed. Observation lasted up to one-year post-KTx in 97 patients (62 treated with ATG and 35 treated with αCD25), with a clinical follow-up of up to three years. One year after KTx, thymic output, or the ability of the thymus to generate new T lymphocytes, was significantly reduced in patients treated with ATG. Furthermore, lymphopoietic progenitors in the bone marrow were declining, with an unfavorably altered l-HPC/m-HPC ratio, and an increase in the presence of highly differentiated T cells, characterized by the CD57^+^/CD28^-^ markers, typical of “aged” lymphocytes. Finally, relative telomere length and telomerase activity in T cells were both reduced. These are emblematic signs of accelerated cellular senescence. Then, ATGs appear to push the immune system of transplant recipients towards a state similar to that of “accelerated aging”: a less efficient thymus, mature T cells that proliferate less and are more difficult to regenerate. Clinically, this could lead to a greater susceptibility to infectious complications and a higher risk of cancer (18). More recently, in the work of Ludvigsen et al, induction with ATG was associated with a significant increase in the risk of PTLD (HR 4.4; 95% CI: 1.8–10.6), while induction with rituximab seemed, on the contrary, to offer relative protection (HR 0.20; 95% CI: 0.03–1.49) (19). Our research group recently published a retrospective single-center study including 930 KTxr followed for a median of 7 (1–19) years. During follow-up, 19% of patients developed at least one post-transplant malignancy, with a mean onset of approximately 83 ± 48 months after surgery. The most frequent tumors were non-melanoma skin cancers (55%), followed by solid tumors (breast, prostate, cervix, lung, urothelial), post-transplant lymphoproliferative disorders (4%), and Kaposi’s disease (2.3%). Furthermore, 32% of patients developed a second malignancy, on average 122 months after transplant. Interestingly, what emerged from the comparison between two immunosuppressive induction regimens is that the regimen including ATG was associated with a significantly higher risk of post-transplant malignancy (HR = 3.4), compared to basiliximab, Furthermore, the onset of cancer occurred on average in 40 months with ATG, compared to 89 months with basiliximab. Regarding survival, patients with cancer had a significantly longer median survival in those who switched to mTOR inhibitors (mTORi) and reduced CNI compared to those who suspended a drug or did not modify their immunosuppressive therapy: 67.4 vs 34.4 months. The same trend was observed in both NMSC and other tumor types (20). The preventive role of mTORi against cancer after renal transplantation was still poorly defined in the real clinical context. In the work of Opelz et al, data from the Collaborative Transplant Study were analyzed on 78146 deceased donor renal transplant recipients (1999–2013), comparing 4279 patients treated with mTORi versus 73867 not treated with these drugs. Propensity score matching was also used to specifically assess the incidence of basal cell and squamous cell carcinomas. The results showed that *de novo* use of mTOR was associated with a significant reduction in the risk of basal cell carcinoma (HR = 0.56), while for squamous cell carcinoma, the risk was reduced but without statistical significance (HR = 0.87). Furthermore, no significant reduction was found for non-skin cancers (HR = 0.94) (21).

Translational research is increasingly focusing on identifying patients at higher risk of developing neoplasms, with growing interest in the study of subclinical markers. In particular, recent attention has turned to microRNAs (miRNAs)as promising subclinical biomarkers for cancer risk (22, 23). In these years, our group is working intensively on mRNAs research, attempting to identify a specific risk pattern for the development of neoplasms post-transplant. In particular, in the paper recently published by Simeoni et. al, miR-210-3p up-regulation, within the cancer-related profile was first found to be associated with non-melanoma skin cancer. In the next future, the knowledge of microRNA, might determine the development of advanced tools for early cancer diagnosis, precise prognosis formulation, and the creation of targeted therapies for KTRs with neoplastic complications (24). All these new evidences can refine deeply the risk stratification and open a clinical direction. Obviously, future evaluations and studies are needed.

## Management of the transplant patient following a cancer diagnosis

Following a cancer diagnosis, the management of kidney transplant recipients (KTxr) involves two key aspects. The first is the adjustment of immunosuppressive therapy, primarily overseen by the transplant nephrologist. This must be closely coordinated with oncology specialists, ensuring strong collaboration in planning and delivering cancer-specific treatments.

### Management of immunosuppressive therapy

Even in the absence of guidelines and high-quality evidence, we can say that a careful reduction in the overall burden of immunosuppression in patients with early- or moderate-stage cancer may represent a reasonable first step in the management of these patients. This intervention should be conducted in consultation with the patient, informing them of any possible adverse effects, and all strategies should be tailored to individual needs. Furthermore, it is important to remember that there are currently no standard guidelines on how immunosuppressive drugs should be modified after a cancer diagnosis. Also, in this case significant importance has been given to mTORi in the past. In the work published by Euvrad et al, 20 renal transplant recipients with at least one cutaneous squamous cell carcinoma previously diagnosed during CNI therapy were enrolled. Patients were assigned to maintain CNI therapy or convert to sirolimus. After two years of follow-up, the sirolimus group showed a significantly reduced risk of developing a new squamous cell carcinoma: 22% of patients versus 39% of the CNI group, with a longer median time to onset (15 vs. 7 months) and a relative risk of 0.56 (95% CI 0.32–0.98). All of this was significant as long as conversion occurred following the first episode of malignancy. The ratio of squamous cell to basal cell carcinomas also improved significantly (from 3.9 to 1.4 with sirolimus, versus 1.8 to 1.0 with CNI). Renal function remained stable in both groups, and no rejection episodes were observed (25). More recently, Dantal et al, extended the observation up to 5 years, confirming that survival free of new cutaneous squamous cell carcinomas was significantly higher in the sirolimus group than in the CNI group, that the number of new skin cancers was significantly lower with sirolimus, and that graft function, patient survival, and graft survival were similar between the two groups. Serious adverse events were reduced between the second and fifth years in the sirolimus group (from a mean of 1.16 to 0.83 per patient) (26). From these last two studies, we can therefore deduce that in KTxr with a history of cutaneous squamous cell carcinoma, conversion from calcineurin inhibitors to mTORi is associated with a significant and long-lasting reduction in the risk of new skin cancers without compromising graft function, representing an effective secondary prevention strategy. In the contest of a need to reduce CNI exposition, belatacept, a CTLA-4-Ig fusion protein that blocks CD80/CD86-CD28 costimulation, dampening naïve and central T-cell activation, is a promising alternative to calcineurin inhibitors by offering effective immunosuppression with reduced (27). However, emerging real-world data have highlighted a growing awareness of infectious and neoplastic complications underscoring the importance of individualized risk assessment and ongoing surveillance (28, 29). Belatacept has prompted extensive research into its association with post-transplant malignancies, particularly post-transplant lymphoproliferative disorder (PTLD), and non-melanoma skin cancers (NMSC) such as cutaneous squamous cell carcinoma (cSCC) (30–32). A pivotal U.S. registry study reported an adjusted hazard ratio of 0.83 for any cancer in KTR treated with Belatacept compared to tacrolimus, suggesting no statistically significant increase in cancer incidence (33).

Numerous clinical trials and registries have been launched to evaluate malignancy profile of Belatacept, including landmark studies like BENEFIT and CTS observational, as well as interventional trials such as BELASBRIDGE, TRANSIBELA, and ATTAIN (34). These studies vary in design, from randomized phase III programs to mechanistic trials, and many explicitly track malignancy and PTLD as endpoints. Some trials have completed with safety data available, while others are ongoing, aiming to clarify cancer risks in specific populations and under different regimen combinations (35). Clinically, the current evidence supports a cautious and individualized approach to Belatacept use. Prior to initiating Belatacept therapy, it is strongly recommended to confirm Epstein-Barr virus (EBV) serostatus, as use in EBV-seronegative patients is associated with an elevated risk of post-transplant lymphoproliferative disorder (PTLD). Even in EBV-seropositive individuals, vigilant monitoring for EBV and cytomegalovirus (CMV) is advised. Clinicians should carefully balance potential oncologic risks against the benefits of belatacept, including nephroprotection and improved metabolic profiles. In patients with recurrent skin cancers or heightened viral susceptibility, combination strategies—such as incorporating mTOR inhibitors—or multidisciplinary evaluation may offer a more tailored and risk-conscious approach.

But how much do these therapeutic modifications impact on patient survival and graft function? This is an interesting point, explored in 2019 by Yang et al. In their retrospective study of 110 patients, it was demonstrated that reducing immunosuppression after cancer diagnosis does not appear to compromise mortality or graft survival, so much so that the only real risk remains the cancer and its treatment. Interestingly, simultaneously reducing both mycophenolate and CNI may pose a risk to the survival of the transplanted kidney. In a context where standardized guidelines are lacking, these results reinforce the idea that management should be personalized (36).

### Oncology therapy management

**Figure 1:**
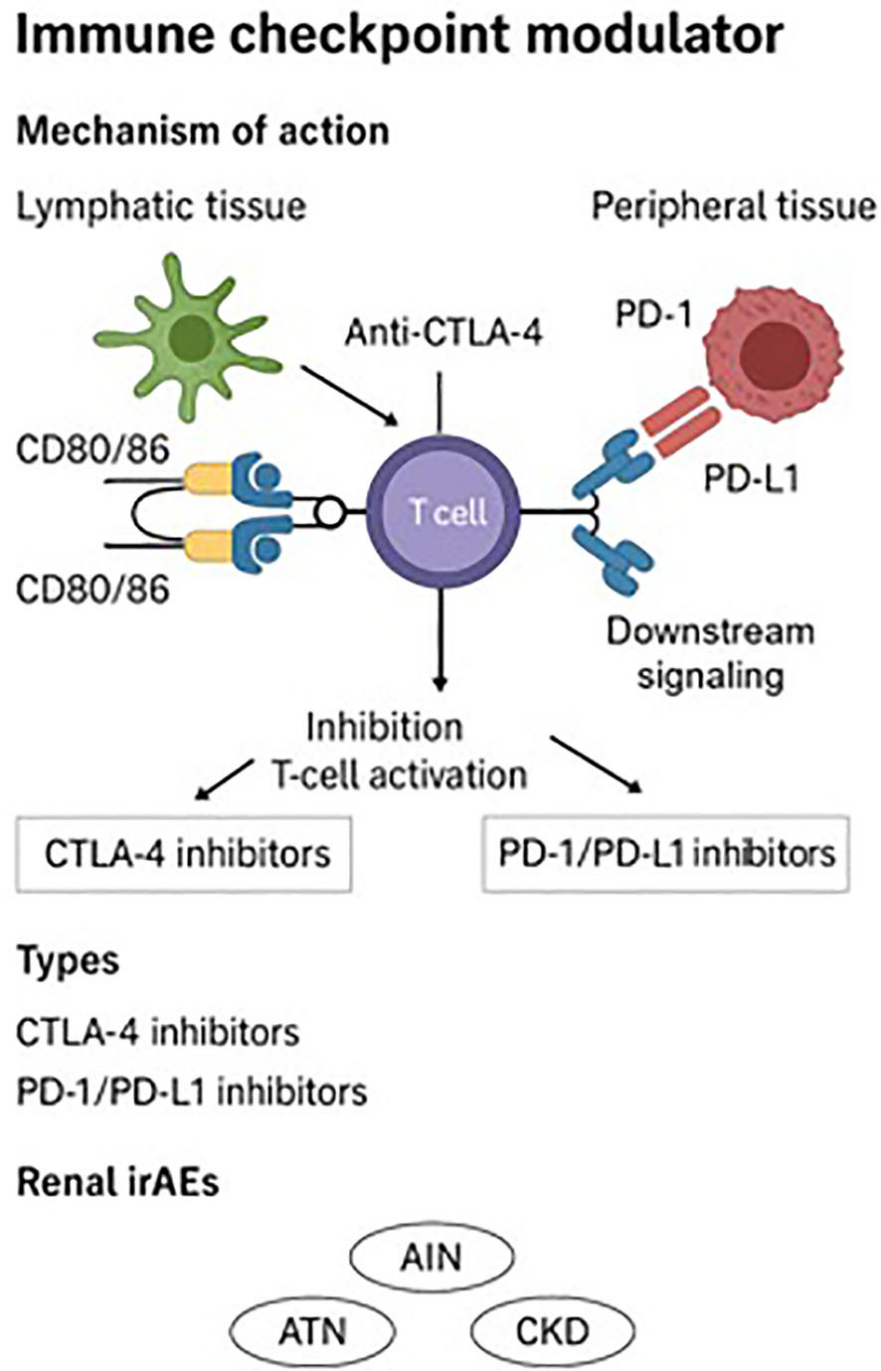
Mechanism of action of checkpoint inhibitors and main renal side effects. AIN, acute interstitial nephritis; ATN, acute tubular necrosis; CKD, chronic kidney disease.

Specific chemotherapy must be tailored to the type of tumor, its severity, and, of course, the patient’s general condition. Considerable attention has been given to the use of immune checkpoint inhibitors, which have shown excellent efficacy in many kidney transplant recipients (KTxr). However, their use carries a notable risk of renal adverse events, particularly acute kidney injury and acute interstitial nephritis, as well as potential glomerular and electrolyte disturbances. Furthermore, in transplant patients, data reported in the literature have previously shown rejection rates related to ICIs use between 40% and 50%, thus substantially limiting the clinical use of these drugs (37, 38). But what determines this risk of rejection? ICIs (anti–PD-1, anti–PD-L1, anti–CTLA-4) remove the physiological brakes on the immune response that are fueled by immunosuppressive therapy (**Figure 1**). This does not occur selectively only against tumor cells, but also reactivates immunity towards the transplanted organ, consequently breaking the tolerance induced by immunosuppression (39). But is it possible to reduce the risk of rejection in patients who are potentially eligible for treatment with ICIs? In recent years, clinical studies have been conducted to evaluate how a possible modification of immunosuppressive therapy could protect patients treated with ICIs from developing rejection. (**Table 1**) These studies have the limitation of having recruited only a few patients. In 2022, Carroll recruited 17 patients with renal transplants and metastatic or incurable solid tumors, treated with nivolumab while maintaining the immunosuppressive therapy unchanged. The rejection rate was 11.7% (2 patients) and the overall response rate was 53% (40). Two years later, a study from the Johns Hopkins University included 8 patients with tumors, treated with nivolumab alone or in combination with ipilimumab, maintaining immunosuppression with tacrolimus (levels 2–5 ng/ml) and prednisone 5 mg/day. Rejection occurred in 25% of cases (2 patients) and the ORR was 33% (41). However, the most comforting data came from the work published by Hanna GJ et al. In this phase I trial of cemiplimab in renal transplant recipients with advanced cutaneous squamous cell carcinoma, the authors adopted a cautious yet innovative approach: patients were gradually converted to an mTORi based therapy combined with pulsed steroids, before starting treatment with cemiplimab (350 mg every three weeks). The results were very encouraging. In fact, no episodes of rejection or graft loss were observed. Serial percentage dd cfDNA values over time remained low and stable, supporting the absence of rejection or graft damage during treatment with cemiplimab. Furthermore, among the 11 evaluable patients, 5 (46%) responded to treatment (including durable responses beyond one year) (42). Certainly, this study marks an important step forward in the integration of ICIs in the renal transplant setting. This model is promising but requires confirmation in larger studies and careful individualization of the immunosuppressive regimen. In current practice, we can assume that ICIs may be considered mainly in selected KTRs with advanced malignancy, meaningful expected oncologic benefit, relatively low immunological risk, stable graft function, and full acceptance of the possibility of graft loss after multidisciplinary discussion.

**Table 1:** Summary of key studies evaluating immune checkpoint inhibitors (ICIs) in kidney transplant recipients with cancer.

Study name	Nivolumab in tx patients	Tacrolimus and ICI	CONTRAC-1
Cancer type	Any cancers (incurable, metastatic solid tumors)	Skin cancers (Melanoma, cSCC, BCC, Merkel cell carcinoma)	cSCC
Transplant	Kidney	Kidney	Kidney
ICI	Nivolumab*	Nivolumab +/- Ipilimumab	Cemiplimab
Immunosuppression	Keep the same dose	Tac (2-5 ng/ml), pred 5 mg/d	mTORi + dynamic pred
Patient #	17	8	12
Rejection	2 (11.7%)	2 (25%)	0 (0%)
ORR (CR + PR)	53%	33%	45%
Registry	ANZCTR CA209-993ISR	NCT03816332	NCT03565783
Primary institution	Royal Adelaide Hospital, multicenter, Australia	Johns Hopkins Hospital, USA	Dana Farber Cancer Institute, USA
Ref.	Lancet Oncol (2022)	J Clin Oncol (2024)	J Clin Oncol (2024)

In the **Table 2**, a practical summary of the pre-transplant evaluation of candidates with prior cancer, risk-stratified post-transplant surveillance, and modulation of immunosuppression and possible use of ICIs after cancer diagnosis is reported.

**Table 2:** Practical approach to three major clinical scenarios in kidney transplant onconephrology.

Clinical scenario	Key questions	Practical assessment	Suggested practical approach	Clinical takeaway
1. Pre-transplant evaluation of candidates with prior cancer	Is the malignancy cured or adequately controlled? What is the realistic risk of recurrence under immunosuppression? Does the expected benefit of transplantation outweigh the oncologic risk?	Cancer type, stage, grade, biology, prior treatments, depth and duration of response, disease-free interval, recurrence risk, comorbidities, life expectancy, and current oncologic eligibility. Multidisciplinary discussion with oncologists/hematologists/urologists is essential, avoiding rigid decisions based solely on fixed waiting times.	Individualized decision-making: immediate listing, delayed listing, or non-listing according to recurrence risk and overall prognosis. When available, tumor-specific prognostic tools may support the assessment, although they are not specifically validated for the transplant setting. A tailored pre- and post-transplant follow-up plan should be documented.	There is no universally validated cancer-specific pre-transplant risk score for kidney transplant candidates; decisions should rely on individualized, multidisciplinary assessment based on tumor biology and prognosis rather than on automatic time-based rules.
2. Risk-stratified post-transplant surveillance	Is the patient at standard or high oncologic risk? Is general population screening sufficient, or is intensified surveillance needed?	History of prior malignancy, skin phototype and actinic damage, previous skin cancers, EBV/HPV/HBV/HCV status, smoking history, age, intensity and duration of immunosuppression, graft function, hereditary syndromes, and family history.	Apply age- and sex-appropriate standard cancer screening, while intensifying surveillance in high-risk patients, particularly for skin cancers and malignancies associated with viral infections or previous cancer history. Preventive strategies should always be included: sun protection, smoking cessation, vaccination when appropriate, infectious disease monitoring, and adherence counseling. Over-screening in low-risk patients should be avoided.	Post-transplant surveillance should not follow a one-size-fits-all model; it should be tailored according to individual oncologic risk, with particular attention to skin cancer prevention and early detection.
3. Modulation of immunosuppression and possible use of ICIs after cancer diagnosis	Is the malignancy potentially curable or mainly palliative? How should the risk of graft loss be balanced against the expected oncologic benefit? Are effective alternatives to ICIs available?	Cancer stage and aggressiveness, available oncologic options, immunologic risk, prior rejection history, graft function, donor-specific antibodies or other biomarkers when available, current immunosuppressive regimen, and fully informed patient preferences.	After cancer diagnosis, a carefully planned reduction of immunosuppressive burden is often considered, usually beginning with the antimetabolite, with possible CNI minimization or switch to mTOR inhibitors in selected cases, especially when there is a plausible oncologic rationale. After a wisely selection of the patients and an adaptation of the immunosuppressive therapy, ICIs might be considered routine therapy in kidney transplant recepients.	

## Conclusions

Cancer is one of the leading causes of morbidity and mortality in kidney transplant patients. Cancer screening programs in this population are, in most cases, derived by analogy from those used in the general population, without fully considering the specificities of chronic immunosuppression. The clinical management of these patients requires a delicate balance between the immunosuppression needed to maintain a functioning graft and the oncologic treatments. These objectives, often seemingly in conflict, require close multidisciplinary collaboration between nephrologists and oncologists to identify the safest and most effective strategy, tailored to the individual patient’s conditions and priorities.
